# Licofelone-DPPC Interactions: Putting Membrane Lipids on the Radar of Drug Development

**DOI:** 10.3390/molecules24030516

**Published:** 2019-01-31

**Authors:** Catarina Pereira-Leite, Daniela Lopes-de-Campos, Philippe Fontaine, Iolanda M. Cuccovia, Cláudia Nunes, Salette Reis

**Affiliations:** 1 LAQV, REQUIMTE, Departamento de Ciências Químicas, Faculdade de Farmácia, Universidade do Porto, Rua de Jorge Viterbo Ferreira, 228, 4050-313 Porto, Portugal; mleite@ff.up.pt (C.P.-L.); dplopes@ff.up.pt (D.L.-d.-C.); cdnunes@ff.up.pt (C.N.); 2 Departamento de Bioquímica, Instituto de Química, Universidade de São Paulo, Av. Prof. Lineu Prestes, 748, 05508-000 São Paulo, Brazil; imcuccov@iq.usp.br; 3 Synchrotron SOLEIL, L’Orme des Merisiers, Saint Aubin, BP48, 91192 Gif-sur-Yvette, France; philippe.fontaine@synchrotron-soleil.fr

**Keywords:** lipid monolayers, DPPC, licofelone, langmuir isotherms, BAM, PM-IRRAS, GIXD

## Abstract

(1) **Background**: Membrane lipids have been disregarded in drug development throughout the years. Recently, they gained attention in drug design as targets, but they are still disregarded in the latter stages. Thus, this study aims to highlight the relevance of considering membrane lipids in the preclinical phase of drug development. (2) **Methods**: The interactions of a drug candidate for clinical use (licofelone) with a membrane model system made of 1,2-dipalmitoyl-*sn*-glycero-3-phosphocholine (DPPC) were evaluated by combining Langmuir isotherms, Brewster angle microscopy (BAM), polarization-modulation infrared reflection-absorption spectroscopy (PM-IRRAS), and grazing-incidence X-ray diffraction (GIXD) measurements. (3) **Results**: Licofelone caused the expansion of the DPPC isotherm without changing the lipid phase transition profile. Moreover, licofelone induced the reduction of DPPC packing density, while increasing the local order of the DPPC acyl chains. (4) **Conclusions**: The licofelone-induced alterations in the structural organization of phosphatidylcholine monolayers may be related to its pharmacological actions. Thus, the combination of studying drug-membrane interactions with the pharmacological characterization that occurs in the preclinical stage may gather additional information about the mechanisms of action and toxicity of drug candidates. Ultimately, the addition of this innovative step shall improve the success rate of drug development.

## 1. Introduction

Drug discovery is primarily based on finding or designing new molecules to target a biomolecule in order to achieve a therapeutic benefit [1]. Proteins are the main biological targets considered in the rational design of novel drugs. However, other biomolecules have been gaining attention in the last decades, with the emergence of DNA and RNA-based gene therapy [2,3] and membrane lipid therapy [4]. Chronologically, lipids were the last biomolecules to be considered as biological targets, for two main reasons: (a) the huge amount of different lipids found in the human body; and (b) powerful lipid profiling techniques, such as high-resolution mass spectrometry, were recently developed [5].

Cell membranes are nowadays considered as a complex, dynamic, and heterogeneous conjunction of lipids, proteins, and carbohydrates, functioning as platforms to control signal transduction. In particular, membrane lipids act either as messengers or as regulators of signaling pathways [4]. The advent of functional lipidomics has been unveiling the specific roles of membrane lipids on diverse pathophysiological processes. Indeed, alterations in lipid composition and structure are currently related to diverse pathological conditions, including inflammation, cancer, neurodegenerative disorders, and metabolic diseases [4,5]. In this context, membrane lipid therapy has emerged with the goal of attaining a therapeutic benefit by designing new compounds able to modify membrane lipid structures or domains [4].

Beyond being considered for the rational design of novel drugs, membrane lipids seem also to be valuable for the preclinical phase of drug development, in which the pharmacokinetic, pharmacodynamic, and toxicity profiles of the selected candidate are fully addressed [1]. In fact, membrane model systems are important tools to predict the lipophilicity of drug candidates, with recognized advantages over the octanol-water system [6,7,8], which is one of the main aspects influencing the pharmacokinetic properties of pharmaceuticals [9]. Regarding pharmacodynamics, membrane lipids can either be the therapeutic target of drugs or may indirectly regulate the activity of membrane proteins [7,8]. Thus, drug-induced alterations in membrane lipids may result in changes in membrane-proteins activity, as already described, for instance, for phospholipase A2 [10]. Finally, the toxicity mechanisms of drugs may also include drug-induced modifications in the structure of membrane lipids, as reported for commercial nonsteroidal anti-inflammatory drugs [7,11] and antibiotics [12].

In this context, this work aims to show the relevance of considering membrane lipids in the preclinical phase of drug development. For that, a candidate for clinical use, namely licofelone, was selected, and its interactions with a membrane model system, viz. lipid monolayers, were evaluated using complementary experimental techniques.

**Figure 1:**
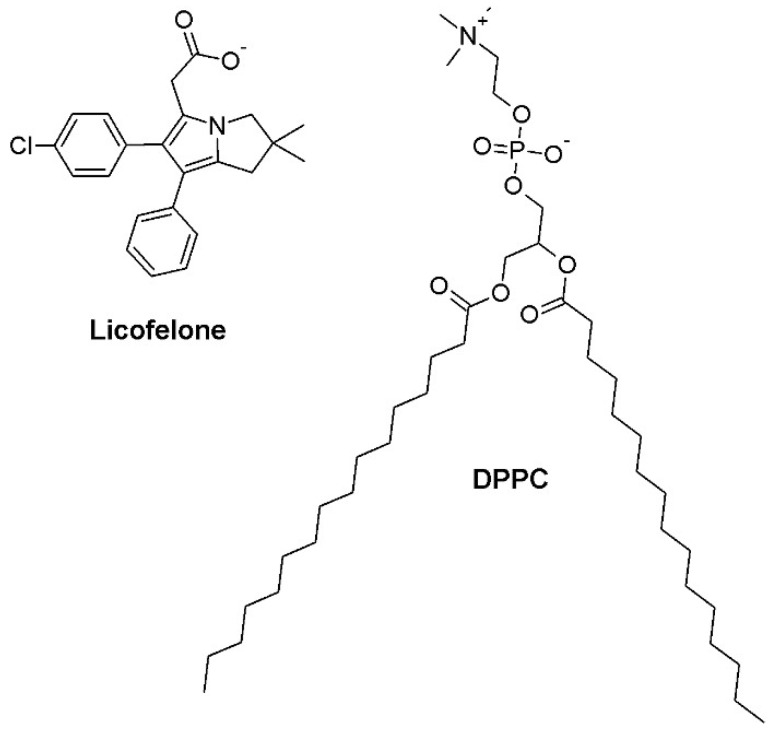
Structural formula of licofelone and 1,2-dipalmitoyl-*sn*-glycero-3-phosphocholine (DPPC).

Licofelone (Figure 1) is a dual cyclooxygenase (COX)/5-lipoxygenase (5-LOX) inhibitor designed to circumvent the toxic actions of nonsteroidal anti-inflammatory drugs, due to the additional inhibition of 5-LOX [13]. Although the released data of preclinical and clinical studies indicate that licofelone has promising efficacy and tolerability profiles [13], this drug was not marketed yet, raising questions about the underlying reasons. In this sense, licofelone was chosen for this study to eventually unravel some unknown membrane lipid-related mechanisms of action and toxicity, which may eventually contribute to clarify the delayed commercialization.

Lipid monolayers made of 1,2-dipalmitoyl-*sn*-glycero-3-phosphocholine (DPPC) at physiological pH (7.4) were chosen as membrane model system. Lipid monolayers are simple and easy-to-make membrane model systems that mimic the lateral interactions occurring in one leaflet of the phospholipid bilayer of cell membranes [14]. Moreover, the lipid packing density and the lateral pressure of biological membranes can be easily mimicked by compressing the lipid monolayer until 30 mN m^−1^ [15]. DPPC was considered an adequate model lipid, since it is a phosphatidylcholine, the main component of mammalian cell membranes [16], and it forms monolayers with defined lipid phase transitions [17,18].

Furthermore, the combination of Langmuir troughs with various detection techniques enables the characterization of lipid monolayers regarding their phase transition behavior by isotherm measurements and Brewster angle microscopy (BAM) [19], as well as the lipid-lipid and lipid-drug molecular interactions through polarization-modulation infrared reflection-absorption spectroscopy (PM-IRRAS) and grazing-incidence X-ray diffraction (GIXD) measurements [14]. In this sense, these techniques were used to evaluate the molecular consequences of adding licofelone to the DPPC monolayer. Overall, the licofelone incorporation in the DPPC monolayer changed the lipid-lipid interactions, which may ultimately be on the basis of the pharmacological actions of this drug candidate for clinical use.

## 2. Results

### 2.1. Langmuir Isotherms

The surface pressure-area per lipid molecule (π-A) isotherms, commonly called Langmuir isotherms, enable studying the phase transition behavior of lipid monolayers upon compression, which is characteristic of each type of lipid [20]. In this sense, the π-A isotherm of the plain DPPC monolayer was firstly characterized to ensure the quality of the data obtained, before evaluating the drug effects.

**Figure 2:**
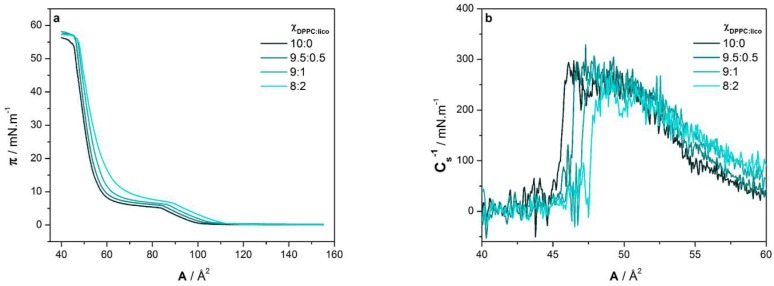
(**a**) Surface pressure-area per lipid molecule (π-A) isotherms of various DPPC:licofelone molar fractions (Χ_DPPC:lico_) at pH 7.4; (**b**) Maximum compressibility modulus (C_s_^−1^) vs area per lipid molecule (A) plot calculated from the obtained π-A isotherms.

The π-A isotherms of DPPC monolayers in the absence and presence of licofelone are shown in Figure 2a. The π-A isotherm of plain DPPC is in agreement with the literature [17,18]. As expected, it was observed upon compression: a) a transition from the gaseous phase to the liquid-expanded (LE) phase at ca. 100 Å^2^; b) a mixed LE and liquid-condensed (LC) phase from 85 to 60 Å^2^ (plateau region); and c) a LC phase from 60 Å^2^ until the monolayer collapses under 45 Å^2^ at a surface pressure of ca. 54 mN m^−1^. By adding licofelone, a concentration-dependent expansion of the DPPC monolayer was observed (Figure 2a). This means that, for the same surface pressure value, the area per lipid molecule increased upon licofelone addition. It is noteworthy that the trend of the licofelone-induced expansion of the DPPC monolayer depends on the surface pressure (Supplementary material, Figure S1). The expansion occurred exponentially with drug concentration for smaller values of surface pressure, while the trend became linear as surface pressure increased. This result indicates that licofelone is gradually squeezed out of the monolayer upon compression, as smaller effects were observed by increasing surface pressure.

To better assess the effect of adding licofelone to the DPPC monolayer, various parameters were calculated from the obtained π-A isotherms, namely the minimum area per lipid molecule (A_min_), the area per lipid molecule at 30 mN m^−1^ (A_30_) and the maximum compressibility modulus (C_s_^−1^). The results are shown in Table 1. A_min_ and A_30_ were determined by fitting a linear regression to the isotherm under maximum packing conditions (A_min_) or around 30 mN m^−1^ (A_30_) and extrapolating it to zero surface pressure [21]. C_s_^−1^ describes the proportional relationship between the surface pressure increase and the area per lipid decrease. Hence, it was calculated from the π-A isotherms, as follows [22]:

(1) $$C_{s}^{-1}=-A\left(d\pi /\mathrm{dA}\right).$$

Licofelone induced a more pronounced increase of A_30_ than A_min_ (Table 1), suggesting, once again, that the drug is partially squeezed out of the monolayer at higher surface pressures. This result is also in line with the π_collapse_ data (Table 1), as no significant alterations were observed for this parameter, confirming the partial exclusion of licofelone from the DPPC monolayer. Concerning the maximum C_s_^−1^ (Figure 2b and Table 1), no significant differences were observed in the presence of licofelone, meaning that the drug did not markedly modify the elastic properties of the DPPC monolayer.

### 2.2. Brewster Angle Microscopy

The morphological alterations induced upon compression of the DPPC monolayer, in the absence and presence of licofelone, were followed by Brewster angle microscopy (BAM). This technique has the remarkable advantage of enabling the visualization of condensed domains of lipid monolayers without introducing external probes. This is possible once condensed phases display nonzero reflectance of a parallel polarized laser beam with the Brewster angle of incidence [19].

**Figure 3:**
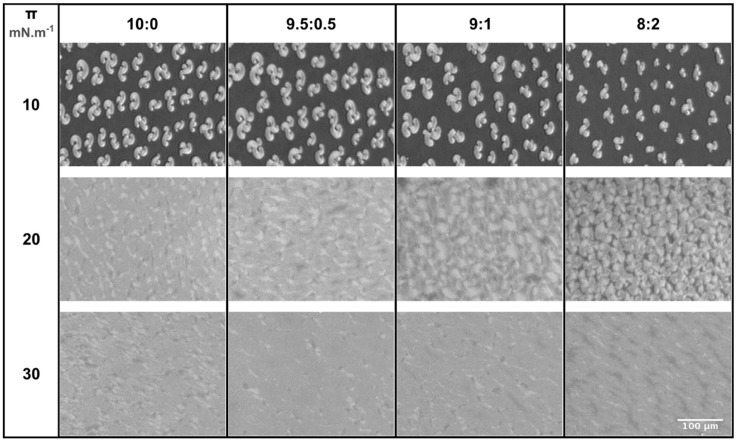
Brewster angle microscopy (BAM) images of Langmuir monolayers containing various DPPC:licofelone molar fractions (10:0, 9.5:0.5, 9:1, and 8:2) at pH 7.4 according to the surface pressure (10, 20, and 30 mN m^−1^). Scale bar is the same for all images.

The BAM images obtained with the plain DPPC monolayer (Figure 3) are in line with the literature [23,24]. By increasing the surface pressure, gray domains with diverse shapes (e.g. dots and bean-shaped domains) appeared on a black matrix (data not shown), and at 10 mN m^−1^ (Figure 3), the domains were in a typical multilobed shape. This result is in agreement with the Langmuir isotherms data (Figure 2a), once the appearance of gray domains indicates that the monolayer was transiting from the LE phase to the LC phase [25]. Afterwards, the DPPC domains got closer and closer, displaying smoother contours, until a uniform gray carpet was formed from ca. 20 mN m^−1^ (Figure 3).

The licofelone-induced alterations in the morphology of the DPPC monolayer were assessed at 10, 20, and 30 mN m^−1^ (Figure 3). At all studied surface pressures, licofelone induced concentration-dependent modifications. At 10 mN m^−1^, the gray domains/black matrix ratio decreased as licofelone concentration increased but no modifications were observed in the domains morphology. This result suggests that licofelone may preferentially interact with the DPPC molecules in the LE phase and may partially rescue DPPC molecules in the more disordered phase, leading to smaller condensed domains. At 20 and 30 mN m^−1^, the licofelone-induced alterations on the gray condensed carpet of DPPC were systematically assessed by calculating the average histograms of the distribution of gray values of multiple images using ImageJ 1.52a software (Supplementary material, Figure S2). At 20 mN m^−1^, various defects in the gray carpet were visible when adding the higher molar fractions of licofelone (9:1 and 8:2). Indeed, interconnected multilobe-shaped domains were still visible for the highest molar fraction of drug (Figure 3). These observations are in line with the calculated histograms (Figure S2a), as for the higher molar fractions of drug the number of pixels obtained at smaller gray values (<150) increased, and these alterations were particularly evident for the 8:2 molar fraction. At 30 mN m^−1^, less imperfections were observed in the condensed carpet of DPPC upon licofelone addition. However, darker regions, i.e. less condensed domains, were observed for the 8:2 molar fraction (Figure 3). The calculated histograms confirmed that less pronounced alterations were induced by licofelone at 30 mN m^−1^, as symmetric peaks were obtained for all DPPC:licofelone molar fractions (Figure S2b). Moreover, these data suggest that the DPPC carpet may be less condensed upon licofelone incorporation, as the peaks are slightly shifted to lower gray values. 

### 2.3. Polarization-Modulation Infrared Reflection-Absorption Spectroscopy

The interactions of licofelone with the DPPC monolayer were evaluated by polarization-modulation infrared reflection-absorption spectroscopy (PM-IRRAS). The drug-induced changes in the wavenumbers of the molecular vibration of the phosphate, carbonyl (CO), and methylene (CH_2_) groups of DPPC, at the surface pressures of 10 and 30 mN m^−1^, were analyzed to retrieve information about the hydration and the conformational order of the lipid monolayer [26]. 

**Figure 4:**
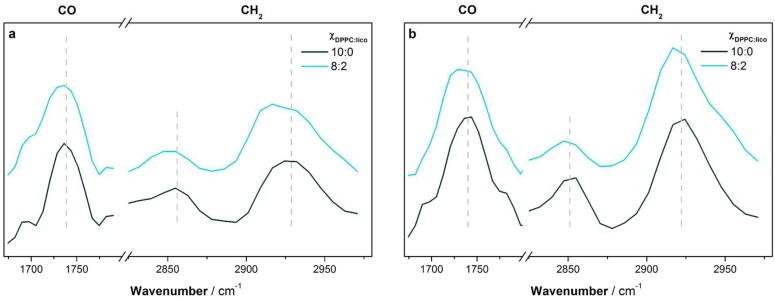
Polarization–modulation infrared reflection–absorption spectroscopy (PM-IRRAS) spectra in the molecular vibration frequency of the carbonyl (CO) and the methylene (CH_2_) groups of the DPPC monolayer at pH 7.4 in the absence (Χ_DPPC:lico_ 10:0) and in the presence of licofelone (Χ_DPPC:lico_ 8:2) at (**a**) 10 mN m^−1^ and (**b**) 30 mN m^−1^. Dashed lines are guides to the eye.

The wavenumbers of the vibrational bands of the asymmetric phosphate, carbonyl, and symmetric and asymmetric methylene groups of the plain DPPC monolayer were in line with the literature [26], namely ca. 1260, 1740, 2854, and 2924 cm^−1^, respectively. The intensity of the CO and CH_2_ vibrational bands increased upon compression (Figure 4), because the decrease of the area per lipid molecule leads to an augment of the signal arriving at the detector, as previously reported for phospholipid monolayers [26].

The effect of the highest molar fraction of licofelone on the PM-IRRAS spectra of the DPPC monolayer was evaluated at 10 and 30 mN m^−1^. This molar fraction was chosen as it caused the most pronounced alterations in the monolayer (Figure 2 and Figure 3), and the results are presented in Figure 4. The phosphate bands were not plotted as no alterations were observed at both surface pressures. On the other hand, both the CO and the CH_2_ vibrational bands were shifted to lower wavenumbers upon licofelone addition. It is noteworthy that the licofelone effects were similar at both surface pressures studied. This result probably reflects the fact that the lipid monolayer was in the LC phase at both 10 and 30 mN m^−1^, irrespective to drug incorporation (Figure 2a). The observed shift of the CO vibrational band suggests that the carbonyl group of DPPC became more hydrated (i.e., more H-bonds formation) in the presence of licofelone [21,26]. Moreover, the decrease of the CH_2_ vibrational frequencies indicates that licofelone promoted an increment in the conformational order of the DPPC monolayer, for instance, by reducing the number of *gauche* rotamers in the acyl chains of DPPC and/or increasing the monolayer packing [21,26].

### 2.4. Grazing-Incidence X-Ray Diffraction

Grazing-incidence X-ray diffraction (GIXD) studies were used to complement the previous results, once they provide information about changes in the structural arrangements and tilt angle variations of phospholipids within a monolayer [27,28]. This is possible due to the existence of an evanescent wave that travels along the air/water interface upon an almost completely reflection of the incident beam [27,28]. GIXD measurements were only performed at 30 mN m^−1^, once similar effects were observed for licofelone at 10 and 30 mN m^−1^ in PM-IRRAS experiments, and this is the lateral pressure of membrane lipids of cell membranes [15].

**Figure 5:**
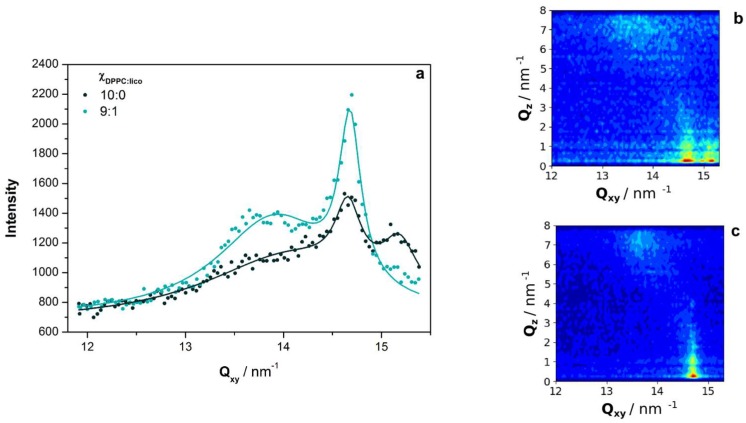
(**a**) Grazing-incidence X-ray diffraction (GIXD) patterns of the DPPC monolayer at pH 7.4 in the absence (Χ_DPPC:lico_ 10:0) and in the presence of licofelone (Χ_DPPC:lico_ 9:1) at 30 mN m^−1^. The corresponding Q_xy_-Q_z_ intensity maps are also presented for (**b**) Χ_DPPC:lico_ 10:0 and (**c**) Χ_DPPC:lico_ 9:1.

Three Bragg peaks were measured in the diffraction patterns of plain DPPC at pH 7.4 and 30 mN m^−1^ (Figure 5a), as previously described [12]. Two out of the three Bragg peaks were in-plane (Figure 5b), indicating that DPPC domains with different structural arrangements coexist in the monolayer. DPPC molecules were organized in a rectangular lattice structure with tilted chains, or in an untilted hexagonal lattice. As the rectangular and tilted lattice is usually described for the DPPC monolayer prepared in various subphases [29,30], domains with hexagonal lattice were considered to be present in the regular rectangular lattice, and the unit cell of the latter was selected for simplicity.

From the diffraction patterns, different parameters were determined from the first-order Bragg peaks, namely the lattice repeat distances (*d*), the correlation lengths (*ξ*), and the acyl chains tilt angle (*θ*). Traditionally, the first-order peak indexations are 1$\bar{1}$ (out-of-plane) and 02 (in-plane) for the tilted rectangular lattice, and 10 (in-plane) for the untilted hexagonal lattice [28]. From the Q_xy_-Q_z_ intensity map (Figure 5b), it is possible to conclude that the acyl chains of DPPC were tilted toward the Next Neighbor (NN-tilt), as 1$\bar{1}$ (out-of-plane) and 02 (in-plane) peaks are present [28]. Thus, the tilt angle values (*θ*) were calculated by the following equation:

(1) $$Q_{z}=Q_{xy}\cos\left(\psi^{*}\right)\tan\left(\theta \right)$$

where *ψ* is the azimuth angle, which is zero in the case of NN-tilt. Moreover, the lattice parameters (a, *b*) were calculated as follows:

(2) $$a=4\pi /\sqrt{2Q_{{xy}_{1\bar{1}}}^{2}+2Q_{{xy}_{11}}^{2}-2Q_{{xy}_{02}}^{2}}\;,$$

(3) $$b=4\pi /Q_{{xy}_{02}}^{2}.$$

The results are shown in Table 2, and the obtained values for the plain DPPC monolayer are in reasonable agreement with the literature [12,31,32].

The diffraction pattern of DPPC monolayers in the presence of licofelone showed only two Bragg peaks, the 1$\bar{1}$ (out-of-plane) and the 02 (in-plane) peaks (Figure 5a,c), characteristic of the rectangular lattice structure. Thus, the condensed untilted domains with the smallest lattice repeat distance ($d_{10}$ = 4.14 Å), i.e. the hexagonal packing, disappeared (Table 2). This result may partially justify the expansion of the Langmuir isotherms (Figure 2a) toward higher area per lipid. Moreover, the a parameter value of the rectangular lattice structured increased, resulting in higher area per lipid molecule than that obtained with plain DPPC. No significant alterations in the *θ* value was observed upon licofelone addition (Table 2), meaning that the orientation of DPPC acyl chains was not influenced by the drug.

## 3. Discussion

Various experimental techniques were combined to comprehensively characterize the molecular interactions of licofelone with a DPPC monolayer, used as membrane model. First, Langmuir isotherms showed that licofelone caused the expansion of the DPPC monolayer (Figure 2a). This effect has been associated with an intercalation of the compound into the phospholipid monolayer [33] and/or an increase of the monolayer fluidity [34]. Since the elastic properties of the monolayer did not varied significantly (C_s_^−1^ values in Table 1), the monolayer expansion seems to be essentially caused by the drug intercalation. This hypothesis was further confirmed by the PM-IRRAS data once the conformational order of the DPPC acyl chains increased upon licofelone incorporation (Section 2.3), showing that licofelone did not increase the monolayer fluidity.

Despite causing the expansion of the DPPC monolayer, licofelone did not disturb the phase transitions of the DPPC monolayer. The drug only shifted the phase transitions toward higher area per lipid molecule and surface pressures, as revealed by the Langmuir isotherms (Figure 2a) and the BAM images (Figure 3). Indeed, the typical condensed domains observed in the BAM images of plain DPPC were also detected in the presence of licofelone, without significant morphological alterations.

**Figure 6:**
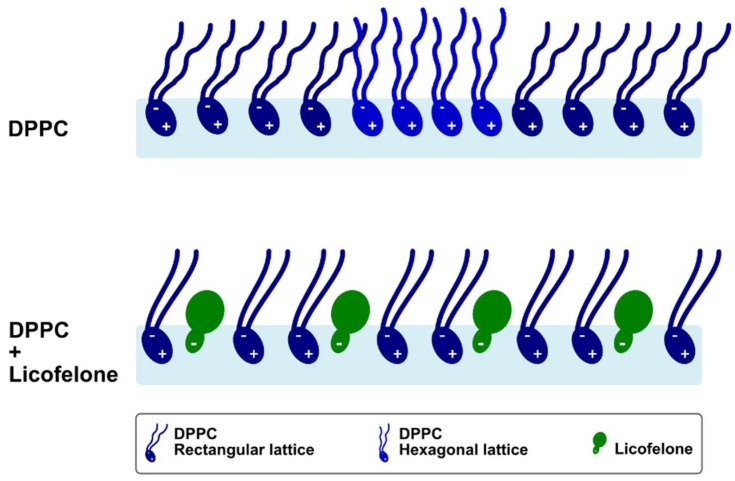
Schematic representation of the licofelone-induced alterations in the DPPC monolayer.

Molecular details about the DPPC-licofelone interactions were retrieved through PM-IRRAS and GIXD experiments. The obtained diffraction patterns revealed that the licofelone-induced expansion of the DPPC isotherm occurs due to the disappearance of highly packed untilted hexagonal domains, as well as the increase of the area per lipid of the tilted rectangular lattice structure. Indeed, the intercalation of licofelone into the DPPC monolayer may disrupt the lipid-lipid interactions, decreasing the DPPC packing density. Lower packing may justify the increase of CO hydration (CO vibrational frequency decreased) upon licofelone addition. Indeed, the CO groups of DPPC have permanent electric dipoles [12], and dipole-dipole interactions with adjacent phospholipids may be disrupted with looser packing, enabling the occurrence of more H-bonds with the aqueous subphase. Despite lowering packing density, the licofelone intercalation also seems to induce a steric constraint on the DPPC acyl chains, which became more ordered (CH_2_ vibrational frequencies decreased) with reduced *trans-gauche* isomerizations. To improve clarity, the main alterations induced by licofelone in the DPPC monolayer are illustrated in Figure 6.

The packing density of the DPPC monolayer may also decrease due to the excess of negative charges introduced in the air-water interface due to the licofelone insertion into the DPPC monolayer. Indeed, the bulk *pK_a_* of licofelone is 4.8 (according to the MarvinSketch calculator from ChemAxon) and its surface *pK_a_* is not expected to increase more than 1 *pK_a_* unit according to literature [35,36,37]. Thus, licofelone has acidic properties (Figure 1), being mainly deprotonated at pH 7.4. Since DPPC is a zwitterionic molecule with an anionic phosphate group and a cationic choline group (Figure 1), it is conceivable that electrostatic repulsions between the phosphate moiety of DPPC and the carboxylate group of licofelone may be a restriction to highly packed domains of DPPC. This hypothesis also explains the fact that licofelone was partially squeezed out of the monolayer under high surface pressures (Section 2.1), as electrostatic repulsions may drive the expulsion of licofelone from the DPPC monolayer.

The structural formula of licofelone (Figure 1) also give insights into the putative orientation of the drug in the DPPC monolayer. It is conceivable that licofelone interacts electrostatically with the choline moiety of DPPC, anchoring the drug at the water interface and facing the aromatic rings toward the air, thereby establishing hydrophobic interactions with the DPPC acyl chains. This orientation justifies the steric constraint induced by licofelone in the DPPC acyl chains. Moreover, it is in line with the literature [38,39,40], regarding the molecular interactions of various anionic anti-inflammatory drugs with phosphatidylcholine molecules.

Overall, this study showed the ability of licofelone to induce alterations into the structural organization of DPPC. These actions may be related to the in vivo pharmacological actions of this pharmaceutical. Regarding anti-inflammatory effects, the licofelone-induced changes in the molecular organization of phosphatidylcholines may result in the indirect inhibition of COX and 5-LOX, its therapeutic targets. Despite being structurally and functionally distinct, both COX isoforms (COX-1 and COX-2) are monotopic membrane proteins located in the endoplasmic reticulum and nuclear envelope [41,42], and COX-2 is usually overexpressed in inflammatory conditions [43]. On the other hand, 5-LOX is a cytoplasmatic enzyme that must bind to the nuclear membrane to become activated [44]. Thus, protein-lipid interactions may modulate the activity of both inflammatory enzymes. Indeed, both COX-2 and 5-LOX have preferential affinity for the fluid domains of biological membranes [44,45]. Thus, the drug intercalation and the drug-induced local ordering in the acyl chains region of phosphatidylcholines may cause a reduction in the activity of these enzymes, favoring the therapeutic actions of licofelone. On the other hand, the reduction of the lipid packing density caused by licofelone may result in gastric toxicity, as described for various acidic nonsteroidal anti-inflammatory drugs [7,40]. In fact, defects in the protective phospholipid layers of the gastric mucosa may facilitate the penetration of noxious agents, such as protons, digestive enzymes and toxins, prompting the occurrence of gastric damage. 

In this sense, the combination of in vitro studies on drug-membrane interactions with in vitro and in vivo pharmacological studies in the preclinical phase of drug development shall provide relevant information about the mechanisms of action and toxicity of drug candidates for clinical use. Before the translation from single studies to drug development, different parameters of these in vitro studies must be optimized to better resemble the conditions found by drugs in their in vivo pathway, such as the type of membrane model, the lipid composition, the composition of the aqueous medium, and the drug concentration. Moreover, the development of high-throughput screening methods would be another important step to implement drug-membrane interactions studies in the laborious process of drug development. Despite the long journey ahead, these in vitro studies may be of utmost importance to avoid pitfalls in the latter stages of drug development, increasing the success rate of the process and reducing the corresponding duration and costs.

## 4. Materials and Methods 

### 4.1. Materials

Licofelone was supplied by Cayman Chemical (Ann Arbor, MI, USA). 1,2-Dipalmitoyl-*sn*-glycero-3-phosphocholine (DPPC) was purchased from Avanti Polar Lipids, Inc. (Alabaster, AL, USA). Trizma^®^ base was obtained from Sigma-Aldrich Co. (St. Louis, MO, USA) and used to prepare the subphase-Tris-HCl buffer (10 mM, pH 7.4). The subphase was prepared using double-deionized water (κ < 0.1 µS cm^−1^) and by adjusting the pH with a hydrochloric acid solution (1 M) after Trizma^®^ base dissolution. Chloroform, methanol, and ethanol were supplied by VWR International S.A.S. (Fontenais-sous-Bois, France). The stock solutions of DPPC and licofelone were prepared in chloroform and methanol, respectively.

### 4.2. Monolayers Preparation

A DPPC solution (1 mg mL^−1^) in chloroform or a DPPC:licofelone solution (9.5:0.5, 9:1, and 8:2 molar fractions) in chloroform:methanol (9:1, *v*/*v*) was dropwise spread using a Hamilton syringe (Bonaduz, Switzerland) onto the subphase (Tris HCl buffer, 10 mM, pH 7.4). The lipid or the lipid:drug monolayers were then equilibrated for 15 min to ensure the organic solvents evaporation.

### 4.3. Langmuir Isotherms

Langmuir isotherm measurements were carried out in a Langmuir balance from KSV NIMA (Helsinki, Finland), using a KN-1006 trough with two symmetrical barriers and a strip of filter paper (Biolin Scientific, Espoo, Finland) as surface pressure sensor. Surface pressure-area per lipid molecule (π-A) isotherms were acquired by compressing the monolayer at a rate of 5 mm min^−1^ at 21 (±1) °C.

Ethanol and double-deionized water (κ < 0.1 µS cm^−1^) were used to properly clean the trough before and after each measurement. The trough was considered clean when the barriers compression did not cause any increase of the basal surface pressure of the subphase (0 mN m^−1^).

### 4.4. Brewster Angle Microscopy

Images of the lipid(:drug) monolayers were captured using a Nanofilm_Ultrabam Brewster angle microscope from Accurion GmbH (Goettingen, Germany) coupled with the Langmuir balance described in the previous section. The images with a lateral resolution of 2 µm were taken at 21 (±1) °C after setting the surface pressure to 10, 20, or 30 mN m^−1^. To improve the contrast of BAM images, the brightness was optimized, and the background was corrected using the Accurion Image 1.1.3 software (Accurion GmbH, Goettingen, Germany).

### 4.5. Polarization-Modulation Infrared Reflection-Absorption Spectroscopy

A PM-IRRAS spectrophotometer from KSV NIMA (Helsinki, Finland) was used for infrared analysis, coupled with a Langmuir balance with a KN-1002 trough with two symmetrical barriers and a strip of filter paper (Biolin Scientific, Espoo, Finland) as surface pressure sensor.

PM-IRRAS measurements were performed using an incident angle of 80° with respect to the normal of the monolayer surface. After optimizing the interferogram to values higher than 6.5, PM-IRRAS spectra were acquired for 300 s at 21 (±1) °C and at 10 and 30 mN m^−1^. The DPPC spectra in the absence and in the presence of licofelone were corrected with the subphase spectra, recorded previously to the lipid(:drug) spreading. 

### 4.6. Grazing-Incidence X-ray Diffraction

GIXD measurements were performed in the SIRIUS beamline at the SOLEIL synchrotron (Gif-sur-Yvette, France). Details about the facility can be found elsewhere [46]. The lipid monolayers were prepared (as described in Section 4.2) in a trough with a helium-flushed sealed chamber. The inert gas was used to reduce the gas scattering and to hamper beam-induced damage to the monolayer. All experiments were performed at 20 (±1) °C.

GIXD measurements were performed using the following settings, as previously described [12]: 10.5 KeV (λ = 0.118 nm) incident X-ray energy; 0.1 × 2 mm^2^ (V × H) beam size; and 1.70 mrad incident angle. This angle is below the critical angle of the air-water interface, enabling the refracted wave to be evanescent. Thus, a 5 nm layer underneath the interface was investigated. The scattered intensity was acquired with high resolution (0.03 nm^−1^ wave vector resolution) using a 1D gas detector fixed on the 2-axis detector arm of the diffractometer, with 2048 channels at 150 mm.

The obtained diffraction patterns were displayed as Q_xy_-Q_z_ intensity maps, which consider the two components of the scattering vector Q, namely Q_xy_ (in-plane component) and Q_z_ (out-of-plane component) [28]. From the vertical integration of the intensity maps along Q_z_, the position, width and intensity of Bragg peaks were determined to characterize the periodic structure of the monolayer. The Q_z_ component was used to calculate the tilt angle of the lipid acyl chains [47]. The Q_xy_ component, in particular the position of the first-order Bragg peaks, were considered to determine the lattice repeat distances ($d=2\pi /Q_{xy}$) [28]. Moreover, the full-width at half-maximum (w) of the first-order Bragg peaks were considered to calculate the correlation lengths (ξ=2/w) [12].

## Figures and Tables

**Table 1:** Minimum area per lipid molecule (A_min_), area per lipid molecule at 30 mN m^−1^ (A_30_), maximum compressibility modulus (C_s_^−1^) ^1^, and surface pressure of collapse (π_collapse_) of the lipid monolayer at pH 7.4 as a function of DPPC:licofelone molar fraction (Χ_DPPC:lico_). Data are presented as mean ± standard deviation (*n* = 3).

Χ_DPPC:lico_	A_min_ (Å^2^)	A_30_ (Å^2^)	C_s_^−1^ (mN m^−^^1^)	π_collapse_ (mN m^−^^1^)
10:0	56 ± 1	57 ± 1	255 ± 21	54 ± 1
9.5:0.5	56 ± 2	57 ± 2	256 ± 20	54 ± 1
9:1	57 ± 1	59 ± 1	241 ± 20	54 ± 2
8:2	59 ± 2	62 ± 2	232 ± 20	55 ± 1

^1^ C_s_^−1^ values stand for the mean ± standard deviation of the maximum plateau region of C_s_^−1^ vs A plots.

**Table 2:** Distances (*d*) ^1^, correlation lengths (*ξ*), lattice parameters (a, b) ^2^ and acyl-chains tilt angle (*θ*) determined from the GIXD patterns of the DPPC monolayer at pH 7.4 and at 30 mN m^−1^, according to lipid:licofelone molar fraction (Χ_DPPC:lico_).

Χ_DPPC:lico_	$d_{1\bar{1}}\;(Å)$	$d_{02}\;(Å)$	$d_{10}\;(Å)$	$\xi_{1\bar{1}}\;(\mathrm{nm})$	$\xi_{02}\;(\mathrm{nm})$	$\xi_{10}\;(\mathrm{nm})$	a (nm)	b (nm)	*θ* (°)
10:0	4.46	4.29	4.14	1.8	12.6	9.2	0.522	0.857	30
9:1	4.53	4.28	-	3.1	15.4	-	0.534	0.856	29

^1^ Standard deviation of lattice repeat distances: ±0.03. ^2^ Lattice parameters for hexagonal phase: a=b= 0.447 nm.

## Supplementary Materials

The following are available online, Figure S1. Variation of the area per lipid molecule (ΔA) according to the licofelone concentration, expressed as DPPC:licofelone molar fraction (10:0, 9.5:0.5, 9:1, 8:2), as a function of surface pressure (π). Figure S2. Average histograms of the distribution of gray values of the BAM images obtained at (a) 20 mN m^−1^ and (b) 30 mN m^−1^, according to the DPPC:licofelone molar fraction (10:0, 9.5:0.5, 9:1, 8:2).

## References

[1] Blass B.E., Blass B.E. (2015). Chapter 1-Drug Discovery and Development: An Overview of Modern Methods and Principles. Basic Principles of Drug Discovery and Development.

[2] Wang D., Gao G. (2014). State-of-the-art human gene therapy: Part II. Gene therapy strategies and clinical applications. Discov. Med..

[3] Burnett J.C., Rossi J.J. (2012). RNA-based Therapeutics- Current Progress and Future Prospects. Chem. Biol..

[4] Escriba P.V., Busquets X., Inokuchi J., Balogh G., Torok Z., Horvath I., Harwood J.L., Vigh L. (2015). Membrane lipid therapy: Modulation of the cell membrane composition and structure as a molecular base for drug discovery and new disease treatment. Prog. Lipid Res..

[5] Sethi S., Brietzke E. (2017). Recent advances in lipidomics: Analytical and clinical perspectives. Prostaglandins Other Lipid Mediators.

[6] Magalhaes L.M., Nunes C., Lucio M., Segundo M.A., Reis S., Lima J.L.F.C. (2010). High-throughput microplate assay for the determination of drug partition coefficients. Nat. Protoc..

[7] Pereira-Leite C., Nunes C., Reis S. (2013). Interaction of nonsteroidal anti-inflammatory drugs with membranes: In vitro assessment and relevance for their biological actions. Prog. Lipid Res..

[8] Lopes D., Jakobtorweihen S., Nunes C., Sarmento B., Reis S. (2017). Shedding light on the puzzle of drug-membrane interactions: Experimental techniques and molecular dynamics simulations. Prog. Lipid Res..

[9] Blass B.E., Blass B.E. (2015). Chapter 5-Medicinal Chemistry. Basic Principles of Drug Discovery and Development.

[10] Gaspar D., Lucio M., Rocha S., Lima J.L.F.C., Reis S. (2011). Changes in PLA(2) activity after interacting with anti-inflammatory drugs and model membranes: Evidence for the involvement of tryptophan residues. Chem. Phys. Lipids.

[11] Pereira-Leite C., Nunes C., Grahl D., Bozelli J.C., Schreier S., Kamma-Lorger C.S., Cuccovia I.M., Reis S. (2018). Acemetacin-phosphatidylcholine interactions are determined by the drug ionization state. Phys. Chem. Chem. Phys..

[12] Lopes D., Nunes C., Fontaine P., Sarmento B., Reis S. (2017). Proof of pore formation and biophysical perturbations through a 2D amoxicillin-lipid membrane interaction approach. Biochim. Biophys. Acta.

[13] Pereira-Leite C., Nunes C., Jamal S.K., Cuccovia I.M., Reis S. (2017). Nonsteroidal Anti-Inflammatory Therapy: A Journey Toward Safety. Med. Res. Rev..

[14] Stefaniu C., Brezesinski G., Möhwald H. (2014). Langmuir monolayers as models to study processes at membrane surfaces. Adv. Colloid Interface Sci..

[15] Peetla C., Stine A., Labhasetwar V. (2009). Biophysical interactions with model lipid membranes: Applications in drug discovery and drug delivery. Mol. Pharm..

[16] Van Meer G., Voelker D.R., Feigenson G.W. (2008). Membrane lipids: Where they are and how they behave. Nat. Rev. Mol. Cell Biol..

[17] Deleu M., Paquot M., Nylander T. (2005). Fengycin interaction with lipid monolayers at the air-aqueous interface-implications for the effect of fengycin on biological membranes. J. Colloid Interface Sci..

[18] Kouzayha A., Besson F. (2005). GPI-alkaline phosphatase insertion into phosphatidylcholine monolayers: Phase behavior and morphology changes. Biochem. Biophys. Res. Commun..

[19] Vollhardt D., Fainerman V.B. (2010). Characterisation of phase transition in adsorbed monolayers at the air/water interface. Adv. Colloid Interface Sci..

[20] Kaganer V.M., Möhwald H., Dutta P. (1999). Structure and phase transitions in Langmuir monolayers. Rev. Mod. Phys..

[21] Alves A.C., Nunes C., Lima J., Reis S. (2017). Daunorubicin and doxorubicin molecular interplay with 2D membrane models. Colloids Surf., B.

[22] Wang Z., Yang S. (2009). Effects of fullerenes on phospholipid membranes: A langmuir monolayer study. ChemPhysChem.

[23] McConlogue C.W., Vanderlick T.K. (1997). A Close Look at Domain Formation in DPPC Monolayers. Langmuir.

[24] Pinheiro M., Arede M., Giner-Casares J.J., Nunes C., Caio J.M., Moiteiro C., Lucio M., Camacho L., Reis S. (2013). Effects of a novel antimycobacterial compound on the biophysical properties of a pulmonary surfactant model membrane. Int. J. Pharm..

[25] Castro C.M., Pinheiro M., Lucio M., Giner-Casares J.J., Camacho L., Lima J.L., Reis S., Segundo M.A. (2013). Insights about alpha-tocopherol and Trolox interaction with phosphatidylcholine monolayers under peroxidation conditions through Brewster angle microscopy. Colloids Surf., B.

[26] Mendelsohn R., Mao G., Flach C.R. (2010). Infrared reflection–absorption spectroscopy: Principles and applications to lipid–protein interaction in Langmuir films. Biochim. Biophys. Acta, Biomembr..

[27] Stefaniu C., Brezesinski G. (2014). X-ray investigation of monolayers formed at the soft air/water interface. Curr. Opin. Colloid Interface Sci..

[28] Stefaniu C., Brezesinski G. (2014). Grazing incidence X-ray diffraction studies of condensed double-chain phospholipid monolayers formed at the soft air/water interface. Adv. Colloid Interface Sci..

[29] Miller C.E., Busath D.D., Strongin B., Majewski J. (2008). Integration of ganglioside GT1b receptor into DPPE and DPPC phospholipid monolayers: An X-ray reflectivity and grazing-incidence diffraction study. Biophys. J..

[30] Neville F., Cahuzac M., Konovalov O., Ishitsuka Y., Lee K.Y.C., Kuzmenko I., Kale G.M., Gidalevitz D. (2006). Lipid Headgroup Discrimination by Antimicrobial Peptide LL-37: Insight into Mechanism of Action. Biophys. J..

[31] Hąc-Wydro K., Flasiński M., Broniatowski M., Dynarowicz-Łątka P., Majewski J. (2011). Properties of β-sitostanol/DPPC monolayers studied with Grazing Incidence X-ray Diffraction (GIXD) and Brewster Angle Microscopy. J. Colloid Interface Sci..

[32] Gzyl-Malcher B., Filek M., Brezesinski G. (2011). Mixed DPPC/DPTAP Monolayers at the Air/Water Interface: Influence of Indolilo-3-acetic Acid and Selenate Ions on the Monolayer Morphology. Langmuir.

[33] Bialkowska K., Bobrowska-Hagerstrand M., Hagerstrand H. (2001). Expansion of phosphatidylcholine and phosphatidylserine/phosphatidylcholine monolayers by differently charged amphiphiles. Z. Naturforsch. C.

[34] Nobre T.M., Pavinatto F.J., Caseli L., Barros-Timmons A., Dynarowicz-Łątka P., Oliveira O.N. (2015). Interactions of bioactive molecules & nanomaterials with Langmuir monolayers as cell membrane models. Thin Solid Films.

[35] Ariga K., Nakanishi T., Hill J.P., Shirai M., Okuno M., Abe T., Kikuchi J. (2005). Tunable pK of amino acid residues at the air-water interface gives an L-zyme (langmuir enzyme). J. Am. Chem. Soc..

[36] Wellen B.A., Lach E.A., Allen H.C. (2017). Surface pKa of octanoic, nonanoic, and decanoic fatty acids at the air-water interface: Applications to atmospheric aerosol chemistry. Phys. Chem. Chem. Phys..

[37] Yang H., Imanishi Y., Harata A. (2015). Estimating pH at the Air/Water Interface with a Confocal Fluorescence Microscope. Anal. Sci..

[38] Nunes C., Brezesinski G., Pereira-Leite C., Lima J.L.F.C., Reis S., Lucio M. (2011). NSAIDs Interactions with Membranes: A Biophysical Approach. Langmuir.

[39] Boggara M.B., Mihailescu M., Krishnamoorti R. (2012). Structural association of nonsteroidal anti-inflammatory drugs with lipid membranes. J. Am. Chem. Soc..

[40] Lichtenberger L.M., Zhou Y., Jayaraman V., Doyen J.R., O’Neil R.G., Dial E.J., Volk D.E., Gorenstein D.G., Boggara M.B. (2012). Insight into NSAID-induced membrane alterations, pathogenesis and therapeutics: Characterization of interaction of NSAIDs with phosphatidylcholine. Biochim. Biophys. Acta Mol. Cell Biol. Lipids.

[41] Smith W.L., DeWitt D.L., Garavito R.M. (2000). Cyclooxygenases: Structural, cellular, and molecular biology. Annu. Rev. Biochem..

[42] Smith W.L., Urade Y., Jakobsson P.J. (2011). Enzymes of the Cyclooxygenase Pathways of Prostanoid Biosynthesis. Chem. Rev..

[43] Conaghan P.G. (2012). A turbulent decade for NSAIDs: Update on current concepts of classification, epidemiology, comparative efficacy, and toxicity. Rheumatol. Int..

[44] Pande A.H., Qin S., Tatulian S.A. (2005). Membrane fluidity is a key modulator of membrane binding, insertion, and activity of 5-lipoxygenase. Biophys. J..

[45] Ackerman W.E. t., Robinson J.M., Kniss D.A. (2005). Despite transcriptional and functional coordination, cyclooxygenase-2 and microsomal prostaglandin E synthase-1 largely reside in distinct lipid microdomains in WISH epithelial cells. J. Histochem. Cytochem..

[46] Fontaine P., Ciatto G., Aubert N., Goldmann M. (2014). Soft Interfaces and Resonant Investigation on Undulator Source: A Surface X-ray Scattering Beamline to Study Organic Molecular Films at the SOLEIL Synchrotron. Sci. Adv. Mater..

[47] Lucio M., Bringezu F., Reis S., Lima J.L.F.C., Brezesinski G. (2008). Binding of nonsteroidal anti-inflammatory drugs to DPPC: Structure and thermodynamic aspects. Langmuir.

