# Supplementary material for: Licofelone-DPPC Interactions: Putting Membrane Lipids on the Radar of Drug Development
Source: Molecules. 2019 Jan 31;24(3):516. doi: 10.3390/molecules24030516 (PMC6384739; doi:10.3390/molecules24030516)
Supplement: Supplementary file 1 [file molecules-24-00516-s001.pdf]

*Supplementary materials*

# Licofelone-DPPC Interactions: Putting Membrane Lipids on the Radar of Drug Development

**Catarina Pereira-Leite [1,2], Daniela Lopes-de-Campos [1], Philippe Fontaine [3], Iolanda M. Cuccovia [2], Cláudia Nunes [1] and Salette Reis [1,*]**

[1] LAQV, REQUIMTE, Departamento de Ciências Químicas, Faculdade de Farmácia, Universidade do Porto, Rua de Jorge Viterbo Ferreira, 228, 4050-313 Porto, Portugal; mleite@ff.up.pt (C.P.-L.); dplopes@ff.up.pt (D.L.-d.-C.); cdnunes@ff.up.pt (C.N.)

[2] Departamento de Bioquímica, Instituto de Química, Universidade de São Paulo, Av. Prof. Lineu Prestes, 748, 05508-000 São Paulo, Brazil; imcuccov@iq.usp.br (I.M.C.)

[3] Synchrotron SOLEIL, L'Orme des Merisiers, Saint Aubin, BP48, 91192, Gif-sur-Yvette, France; philippe.fontaine@synchrotron-soleil.fr (P.F.)

* Correspondence: shreis@ff.up.pt; Tel.: +351-220-428-672

## 1. Licofelone-induced expansion of the DPPC monolayer

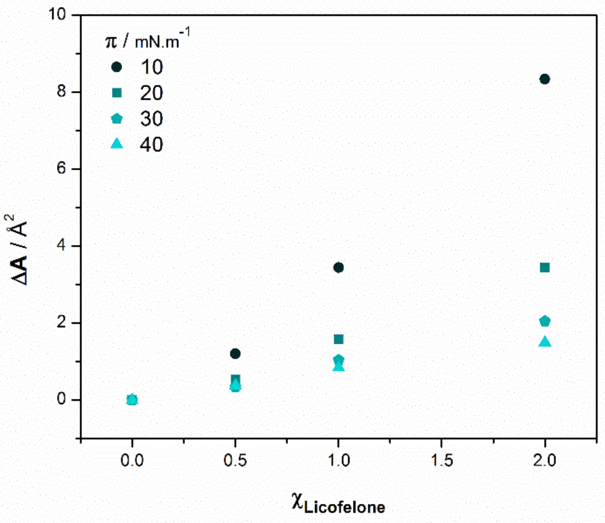


**Figure S1.** Variation of the area per lipid molecule (ΔA) according to the licofelone concentration, expressed as DPPC:licofelone molar fraction (10:0, 9.5:0.5, 9:1, 8:2), as a function of surface pressure (π).

## 2. Licofelone-induced alterations in the gray condensed carpet of DPPC

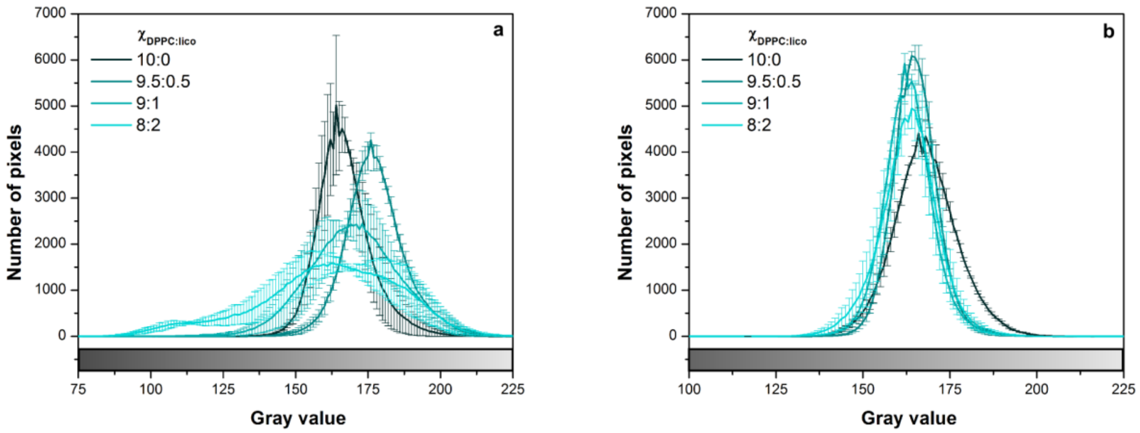


**Figure S2.** Average histograms of the distribution of gray values of the BAM images obtained at (**a**) 20 mN $m^{-1}$ and (**b**) 30 mN $m^{-1}$, according to the DPPC:licofelone molar fraction (10:0, 9.5:0.5, 9:1, 8:2).
